# An Internet-Based Therapeutic Tool for American Indian/Alaska Native Adults With Posttraumatic Stress Disorder: User Testing and Developmental Feasibility Study

**DOI:** 10.2196/13682

**Published:** 2019-11-13

**Authors:** Vanessa Y Hiratsuka, Laurie Moore, Jaedon P Avey, Lisa G Dirks, Barbara D Beach, Denise A Dillard, Douglas K Novins

**Affiliations:** 1 Research Department Southcentral Foundation Anchorage, AK United States; 2 Centers for American Indian and Alaska Native Health University of Colorado Anschutz Medical Campus Aurora, CO United States; 3 Cherokee Nation Behavioral Health Tahlequah, OK United States

**Keywords:** formative research, posttraumatic stress disorder, Web-based intervention, Indians, North American

## Abstract

**Background:**

Posttraumatic stress disorder (PTSD) is a major public health concern among American Indian and Alaska Native populations. Primary care clinics are often the first point of contact for American Indian and Alaska Natives seeking health care and are feasible locations for trauma-focused interventions.

**Objective:**

Web-based therapeutic interventions have the potential to reduce PTSD symptoms by offering psychoeducation and symptom self-management tools. We investigated the feasibility of a culturally adapted Web-based therapeutic intervention in two American Indian and Alaska Native–serving primary care sites. We developed and tested a self-guided Web-based therapeutic intervention aimed at improving knowledge and awareness of, and provision of guidance, support, and symptom-management for, PTSD symptoms.

**Methods:**

A community-based participatory research process was used to refine adaptations to the veteran’s administration’s PTSD Coach Online, to develop new content, and to guide and interpret the results of the feasibility pilot. This process resulted in a 16-guide intervention *“Health is Our Tradition: Balance and Harmony after Trauma”* website. The feasibility pilot included 24 American Indian and Alaska Natives aged 18 years and older who scored positive on a primary care PTSD screener. Enrolled participants completed a demographic questionnaire, an experience with technology questionnaire, and baseline behavioral health measures. Once measures were complete, research staff described weekly text messages, minimum study expectations for website use, and demonstrated how to use the website. Feasibility measures included self-reported website use, ratings of satisfaction and perceived effectiveness, and website metrics. Feasibility of obtaining measures for an effectiveness trial was also assessed to include behavioral health symptoms and service utilization through self-report instruments and electronic health record queries. Self-reported measures were collected at enrollment and at 6 and 12 weeks post enrollment. Electronic health records were collected from 12 months before study enrollment to 3 months following study enrollment. Changes between enrollment and follow-up were examined with paired *t* tests, analysis of variance or logistic regression, or the Wilcoxon signed rank test for nonnormally distributed data.

**Results:**

The culturally adapted website and associated text message reminders were perceived as satisfactory and effective by participants with no differences by age or gender. The majority of participants (86%, 19/24) reported use of the website at 6 weeks and nearly all (91%, 20/22) at 12 weeks. At 6 weeks, 55% (12/22) of participants reported using the website at the recommended intensity (at least three times weekly), dropping to 36% (8/22) at 12 weeks. Participant use of modules varied from 8% (2/24) to 100% (24/24), with guide completion rates being greater for guides that were only psychoeducational in nature compared with guides that were interactive. There were no significant changes in patterns of diagnoses, screening, medications, or service utilization during exposure to the website.

**Conclusions:**

*“Health is Our Tradition: Balance and Harmony after Trauma”* shows promise for an effectiveness pilot.

## Introduction

### Background

American Indian (AI) and Alaska Native (AN) people experience disproportionally higher rates of acute, chronic, and intergenerational trauma than their non–AI/AN counterparts [1-4], with adverse impacts on physical and behavioral health [1,5-9]. As the frequency of traumatic events is significantly elevated, there is an increased likelihood that an AI/AN individual will experience multiple traumas compared with national samples [2,10-12,13]. Multiple lifetime traumas have additive risks to developing behavioral health disorders such as posttraumatic stress disorder (PTSD). AI/AN people have low rates of access to behavioral health services, especially those that are culturally responsive. Primary care clinics are often the first point of contact for AI/AN people seeking health care and are feasible locations for trauma-focused interventions [14-16].

Given the multitude of medical (eg, chronic pain, irritable bowel syndrome, and autoimmune disorders) [17-19] and behavioral (eg, depression, substance use disorders, eating disorders, somatization disorders, personality disorders, and PTSD) [20-23] health problems that occur following traumatic events, clinicians face multiple challenges in appropriately addressing these conditions and their comorbidities. This is especially critical as some research has suggested that the quality of medical outcomes for individuals with a history of trauma is related to their behavioral health outcomes [24,25]. Although behavioral health outcomes may be particularly important in ensuring good medical outcomes for individuals with a history of trauma, psychotherapeutic treatments for these conditions often involve the use of complex, manualized treatment that many behavioral health clinicians have not been trained to deliver (eg, cognitive processing therapy for PTSD and dialectical behavioral therapy for borderline personality disorder) [26,27]. Not all patient education programs are effective in improving health knowledge and treatment outcomes [28]. Research suggests that, to be effective, patient education programs must be intensive, focused on developing specific skills, and fostering a stronger sense of self-efficacy [28].

Health information technologies (HIT) offer innovative solutions to address patient access to critical health information and to reinforce key components of patient treatment plans. HITs offer multiple communication (eg, websites, apps for mobile phones, and clinical decision support systems) to improve the quality of service delivery [29-31], with interactive HIT shown to increase patient knowledge and related health outcomes [32,33] and reduce hospitalizations and health care costs [34]. Web-based therapeutic interventions (WBTIs) are self- or clinician-guided programs that are developed with the aim of positively improving knowledge, awareness, support, and treatment for health problems at low cost. WBTIs are a promising intervention modality for behavioral health treatment support among AI/AN and other indigenous people who have barriers to effective treatment because of access to care as WBTIs can be culturally tailored to address specific clinical issues and the unique characteristics of patient populations.

### Objectives

We undertook a feasibility pilot to develop and test a self-guided, WBTI aimed at improving knowledge and awareness of, and provision of guidance, support, and symptom-management for, PTSD symptoms. This manuscript describes our examination of the website-based intervention’s feasibility in 2 AI/AN–serving primary care sites—the Cherokee Nation Health Services (CNHS) and Southcentral Foundation (SCF). Feasibility was assessed by website metrics, use reported by participants, and participant ratings of satisfaction and perceived effectiveness. We also assessed the feasibility of querying data from the electronic health record 12 months prior and 3 months after enrollment and obtaining self-reported PTSD and other behavioral health symptoms at enrollment, at 6 weeks (intervention midpoint), and at 12 weeks. Finally, patterns of change in behavioral health symptoms, diagnoses, and service utilization served as an additional feasibility indicator for an effectiveness pilot.

## Methods

### Steering Committee

**Figure 1:**
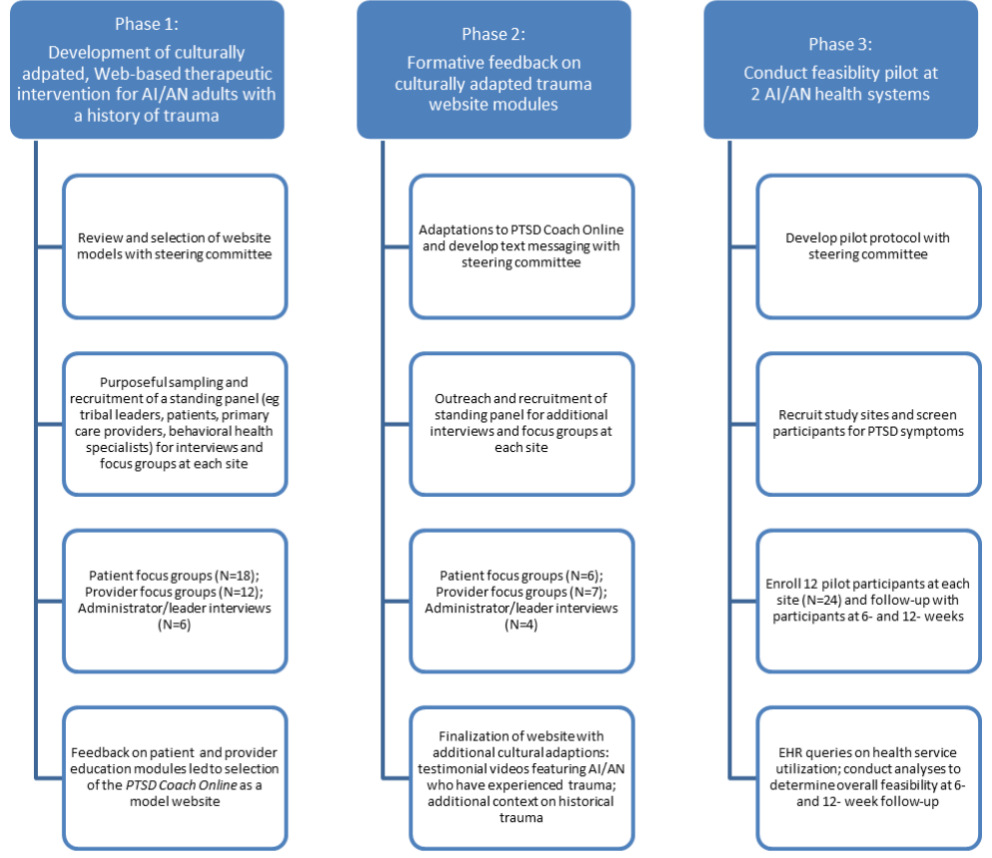
Process for development and implementation of feasibility pilot. AI/AN: American Indian and Alaska Native; PTSD: posttraumatic stress disorder; EHR: electronic health record.

An 11-person steering committee guided each step of the cultural adaptation of the website and associated text messages. A community-based participatory research process was used to refine adaptations and content, and to guide and interpret the results of the feasibility test of the resultant intervention (Figure 1). The steering committee was composed of individuals from the 2 tribal health study sites and the University of Colorado Denver (UCD). Members of the steering committee had multiple roles within their respective organizations. For instance, 5 individuals were clinical providers (3 at SCF, 2 at CNHS, and 1 at UCD), 6 individuals were community members at their study locations (4 at SCF and 2 at CNHS), 1 individual from SCF held an administrative leader role, and 6 individuals had extensive AI/AN health research backgrounds (4 at SCF and 2 at UCD). The committee included the cross-site Principal Investigator and study coordinator from UCD, and a site Principal Investigator at SCF and CNHS. Under advisement of the Steering Committee, we sought broad community feedback on the intervention content at both SCF and CNHS in 2 phases and verified the intervention materials in a second round of qualitative feedback using methods previously implemented by the research team [35]. Following development and AI/AN cultural adaptation of the website, the feasibility pilot was conducted at CNHS and SCF as described further.

### Setting and Participants

The project settings included 2 of the largest AI/AN–serving primary care facilities in the United States: CNHS and SCF. The Cherokee Nation operates a network of 8 health centers and 1 hospital in the tribe’s jurisdictional boundaries, serving more than 130,000 AI patients. CNHS operates the W.W. Hastings Hospital in Tahlequah, Oklahoma, which provides primary care and behavioral health services. SCF is an AN nonprofit health care organization that provides a wide range of health services to over 65,000 AN/AIs from 231 federally recognized tribes in the Anchorage Service Unit. The Anchorage Service Unit is a geographical area extending from the Canadian border on the east to the entire Aleutian Chain and Pribilof Islands on the west, although most SCF patients live near Anchorage, Alaska. SCF operates the Anchorage Native Primary Care Center, which provides primary care and behavioral health services. Each site provides both primary care and behavioral health specialty services and has behavioral health staff embedded within primary care who screen for depression and substance use. The Centers for American Indian and Alaska Native Health at the University of Colorado—Anschutz Medical Campus provided overall coordination between sites. Before data collection, the Alaska Area, Cherokee Nation, and Colorado Multiple Institutional Review Boards approved study procedures. SCF provided tribal approval.

### Cultural Adaptation

Website content was designed to align with national guidelines and local CNHS and SCF preferences for behavioral health interventions. To develop website and text message content, we first completed 2 cycles of 1-hour, semistructured interviews with key stakeholders (providers and clinical administrators) and 2-hour focus groups with providers/administrators and patients in each setting. Leaders and providers were concerned that exposure to website content would be triggering for participants and were concerned about additional health service utilization. Qualitative feedback indicated that a website intervention for PTSD would be welcome with several qualifications, including the emphasis should be on people in context of their community and families, patient modules should be accessible on a website rather than exclusively through mobile technology, and all website information should be consistent with existing clinical recommendations from primary care providers. The adapted content consisted of patient education on the website and the communication of weekly *tips* via text messaging.

On the basis of qualitative feedback, the research team revised the *PTSD Coach Online* [36,37], originally developed by the Veterans Administration to benefit those coping with stress following a traumatic event. The revision process included consultation with our Steering Committee and SCF’s Family Wellness Warriors Initiative (FWWI) [38], an initiative that focuses on providing services for healing from trauma and abuse. The resultant website entitled *Health is Our Tradition: Balance and Harmony after Trauma* included patient education and interactive healing activities (Table 1) [39].

**Figure 2:**
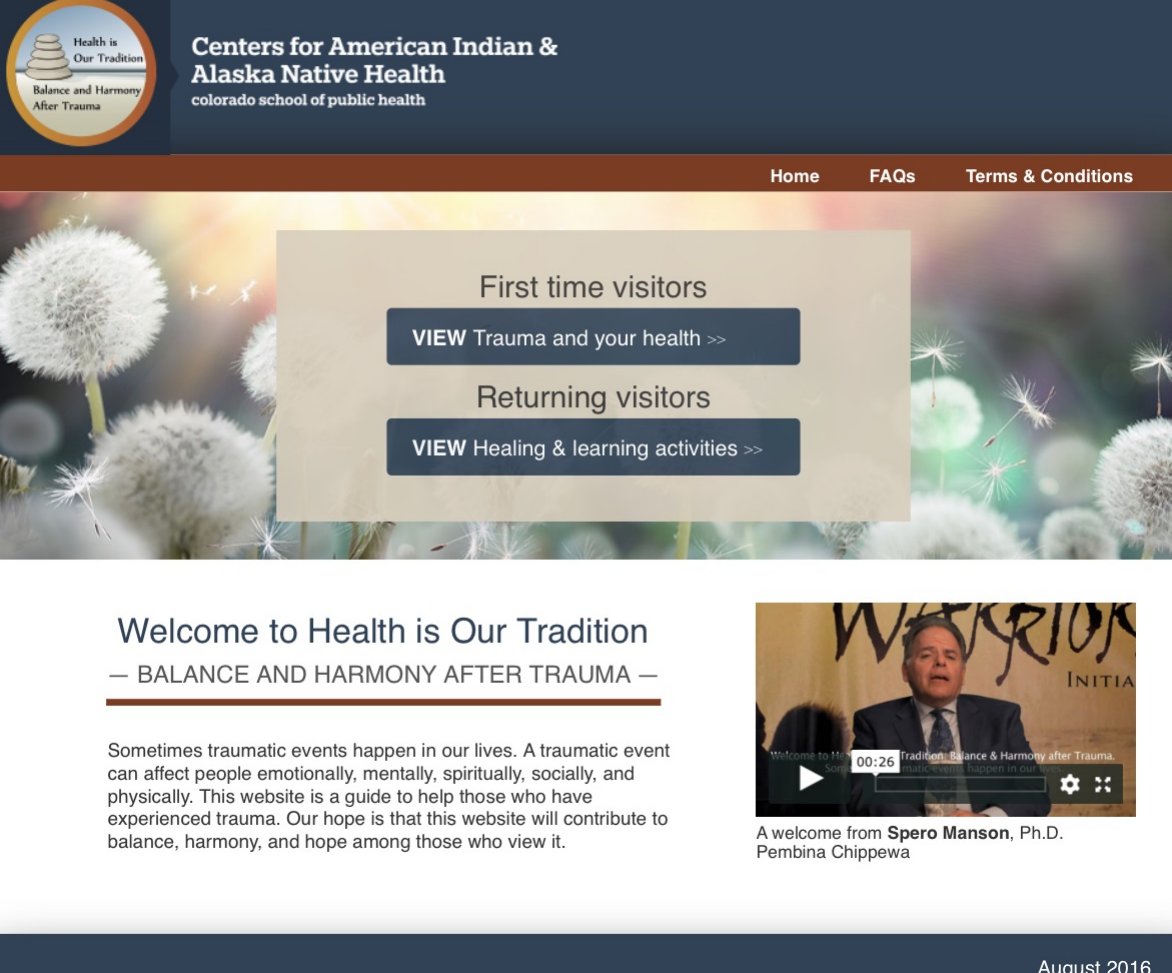
Health is Our Tradition homepage.

The *Health is Our Tradition: Balance and Harmony after Trauma* website included 16 sections referred to as guides [39]. Web page content was either educational only or educational with an interactive activity (eg, drag and drop), an observation/listening activity (eg, audio recorded relaxation exercises), or a written activity (eg, developing a plan for coping with trauma reminders). Video testimonials from AI/AN people who had experienced traumatic events were included in each guide. Web pages used text, programmed interactions, animations, and English language close-captioned videos to present the guide content. Select guides included an audio icon to click for listening to narration of the screen contents. In conjunction with the website, we developed strategic patient communications sent via weekly text-messaged *tips* (Table 2). Following the initial intervention adaptations, we conducted a second cycle of qualitative feedback with information used to further refine the adapted website. A professional website development organization was engaged to develop, test, and deploy the adapted website, whereas the research team planned for testing feasibility of its use in the participating primary care clinics (Figure 2).

**Table 1:** Intervention modules and associated adaptations based on qualitative feedback.

PTSD^a^ Coach Online tools	Health is Our Tradition guides	Description	Adaptations
N/A^b^	Trauma and your Health	Patient education	Content developed from Phase 1 interviews, focus groups, and recommended existing SCF^c^ and CNHS^d^ patient education materials on the impact of trauma on overall health including testimonial videos from AI/AN^e^ people with a history of trauma
Be in the moment	Be in the moment	Guided relaxation and grounding activities	Minor adaptation to add an estimation of activity duration and information before the guided relaxation activity acknowledging that some people may not have a quiet place to listen to the audio activity
Change feelings by changing thoughts	Change feelings by changing thoughts	Educational activities with written exercises to work toward changing negative beliefs about oneself to more helpful positive beliefs	Major adaptation to focus on false beliefs and shame messages common among AI/AN people who have experienced trauma including educational videos narrated by members of the AI/AN community; content developed based on Phase 1 interviews and focus groups, addition of testimonial videos from AI/AN people with a history of trauma
Change how you think about sleep	Change how you think about sleep	Educational activities with interactive content that suggests new ways of thinking about negative thoughts that impair sleep	Minor adaptation to replace the word nightmare with bad dream
Change negative thinking patterns	Change negative thinking patterns	Educational activities with written exercises to work identifying and changing negative thought patterns	No adaptation outside of those listed in Overall
Deal with trauma reminders	Cope with trauma reminders	Educational and guided relaxation activities with written exercises to learn to cope with reminders or triggers related to trauma	Minor adaptation to rename guide from Deal with trauma reminders to Cope with trauma reminders and to add additional examples of triggers
N/A	Develop healthy relationships	Educational and written activities to help identify how trauma may affect interactions with others and to change negative interactions to more positive ones	Content developed from Phase 1 interviews and focus groups and adapted from FWWI^f^ curriculum materials including testimonial videos from AI/AN people with a history of trauma
N/A	Discover your story	Educational and written activities to encourage acceptance and sharing of one’s story that includes traumatic experiences and their effects on resilience and personal growth	Content developed from Phase 1 interviews and focus groups and adapted from FWWI curriculum materials including testimonial videos from AI/AN people with a history of trauma
Form good sleep habits	Form good sleep habits	Educational and interactive activity to learn about good sleep habits	Minor adaptation to replace a drawing of generic sleeping man with one more easily recognized as having AI/AN heritage; changed the drag and drop motion in the interactive activity to a mouse click motion
Identify your values and goals	Identify your values and goals	Educational and written activity to help identify values and goals to guide decisions in life	Minor adaptation in wording of text and omitting the calendar feature
Learn to be assertive	Express your feelings and wants	Educational, written, and checklist activities to foster effective communication skills	Major adaptation to replace all references to assertiveness with expressing oneself and to replace educational and interactive activities with content adapted from FWWI
Notice your thoughts and feelings	Notice your thoughts and feelings	Educational and mindfulness exercises to help user learn to live in the present moment	Minor adaptation to add an estimation of activity duration and information before the mindfulness activities acknowledging that some people may not have a quiet place to listen to the audio activity
Plan something enjoyable	Plan something fun	Educational and interactive activities to help users identify and plan fun activities	Minor adaptation to add or omit suggested activities that are common or uncommon in AI/AN communities; omitted the calendar function
Relax through breathing	Relax through breathing	Animated relaxation activity focused on deep breathing	No adaptation outside of those listed in Overall
Relax through visualization	Relax through visualization	Animated relaxation activity focused on picturing a calm or comfortable scene	Minor adaptation to add an estimation of activity duration and information before the guided relaxation activity acknowledging that some people may not have a quiet place to listen to the audio activity
Relax your body	Relax your body	Animated relaxation activity focused on releasing body tension	No adaptation outside of those listed in Overall

^a^PTSD: Posttraumatic stress disorder.

^b^N/A: not applicable.

^c^SCF: Southcentral Foundation.

^d^CNHS: Cherokee Nation Health Services.

^e^AI/AN: American Indian and Alaska Native.

^f^FWWI: Family Wellness Warriors Initiative.

**Table 2:** Text reminders.

Timing	Text message	Related Health is Our Tradition guide(s)
Week 1	Did you log in to the website yet? If you need our help, call [site research assistant name] at [site phone number].	N/A^a^
Week 2	Have you ever wondered how a traumatic event can affect a person’s health? This information and more can be found in the website guide called Trauma and your health	Trauma and your health
Week 3	Is there one best way to heal from trauma? The answer is no, there are many different things to try. Some may work better for you than others. Go to the guide called How to get back in harmony in the website to find out more.	How to get back in harmony
Week 4	What is a grounding activity and how can it help you feel more safe and stable while healing from trauma? This information and more can be found in the website guide called Be in the moment.	Be in the moment
Week 5	Having trouble falling asleep? The website guides Form good sleep habits and Change how you think about sleep will help you work on sleep problems.	Form good sleep habits; Change how you think about sleep
Week 6	We are looking forward to your midpoint check in visit. Until then, remember to be gentle with yourself. Learning new ways to help yourself heal from trauma can be difficult. Give yourself permission to heal at your own pace and on your own terms.	Identify your values and goals
Week 7	Feeling like you are alone on your healing journey? Throughout the website, there are real people telling their own stories of harm, healing, and their return to wellness. These stories may help you feel less alone with yours.	Discover your story
Week 8	Do you feel stuck with negative thoughts about your past and future? Learn more about ways to change your perspective in the Change feelings by changing thoughts guide.	Change feelings by changing thoughts
Week 9	Doing something you enjoy can help you feel better. Get some great ideas for planning and doing a fun activity in Plan something fun.	Plan something fun
Week 10	Feeling frazzled, stressed, or worried? Try a relaxation activity from any of the following guides: Relax through visualization, Relax your breathing, or Relax your body.	Relax through breathing; Relax through visualization; Relax your body
Week 11	Is there a situation where you feel your voice has been silenced? Working through the activities in the guide called Expressing your feelings and wants may give you a way to break the silence.	Express your feelings and wants
Week 12	We are looking forward to seeing you for your final research visit when we can celebrate the progress you have made toward balance and harmony.	N/A

^a^N/A: not applicable.

### Recruitment and Intervention Procedures

Research staff used lobby-based recruitment at both sites to recruit participants for the intervention. Staff recruited and enrolled 12 participants, at each site, as they waited for their scheduled appointments with a primary care provider. Recruitment occurred in winter 2016 through spring 2017.

Patients expressing interest at the lobby recruitment table were screened by trained staff for study eligibility and completed the informed consent process with eligible individuals. Inclusion criteria included ≥18 years of age, of AI/AN heritage, diagnosis of one or more chronic conditions (ie, heart disease, stroke, diabetes, kidney disease, arthritis, osteoporosis, cancer, asthma, depression, and chronic obstructive pulmonary disease), and 2 or more visits to the primary care clinic in the past 12 months. Exclusionary criteria included lack of access to a text message–capable mobile phone and a computer with an internet connection as well as cellular phone service for the 3 months following enrollment. Ineligible participants were thanked for their participation with a US $10 gift card and returned to the waiting room for their provider appointment.

After completing their primary care provider visit, participants met with a licensed, PhD level psychologist (CNHS) or a Master’s level Behavioral Health Consultant (SCF) to complete the Primary Care—PTSD Screen (PC-PTSD) [40] and an assessment assuring the participant was not currently in crisis. Any participants who were in crisis were counseled by the licensed psychologist/Behavioral Health Consultant and referred to additional behavioral health services, if needed. These participants and those who scored negative on the PC-PTSD were thanked for their participation with a US $10 gift card.

**Figure 3:**
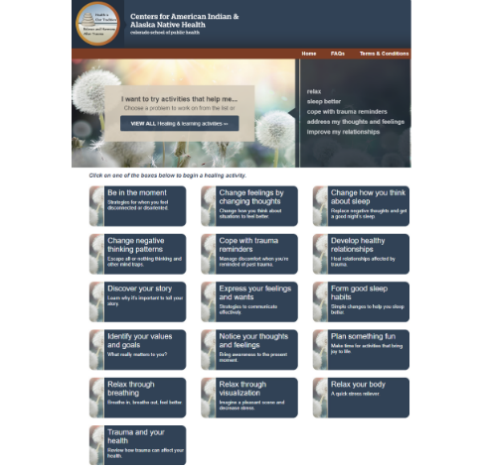
Health is Our Tradition guides.

Participants who were not in crisis and scored positive on the PC-PTSD [39] completed a demographic questionnaire, an experience with technology questionnaire, and the following baseline behavioral health measures: Alcohol Use Disorders Identification Test (AUDIT) [41], Drug Abuse Screening Tool (DAST) [42], AI Symptom Inventory, PTSD Checklist—Civilian Version (PCL-C) [43], Patient Health Questionnaire Depression Scale (PHQ-9) [44], and Patient Health Questionnaire—Somatic, Anxiety, and Depressive Symptoms [45]. Once measures were complete, research staff described the weekly tips (text messages) and the website (Figure 3), including minimum study expectations for its use, and demonstrated how to use the website. Participants were asked to log in and use the website 5-10 min a minimum of 3 times a week over the 12-week intervention period. This expectation was a Steering Committee’s recommendation based on clinical judgment as well as Phase 1 interview and focus group data. Finally, research staff scheduled the 6- and 12-week follow-up visits and thanked participants with a US $60 gift card.

A weekly tip via text message was sent by researchers to participants during the 12-week intervention (Table 2). Text message verbiage was devised to reinforce website use, complement website content, and remind participants about follow-up visits. Research staff also called each participant to remind them of follow-up appointments.

At each follow-up visit (6 weeks and 12 weeks), participants repeated the experience with technology questionnaire, all baseline measures, as well as a use, satisfaction, and perceived effectiveness questionnaire. Upon completion of each follow-up visit, research staff thanked participants and provided them with a $50 gift card. Finally, a qualified staff member, approved at each site to access electronic health records, did so after the last participant completed the 12-week follow-up. Measures from the electronic health record query were collected for the periods extending to 12 months before study enrollment and 3 months following study enrollment.

### Measures

#### Demographic Questionnaire

At baseline, data were collected on participants’ self-reported demographic characteristics. Racial/ethnic heritage was asked using a check all that apply list with the following response categories: AN, AI, white/European-American non-Hispanic, Filipino, other Asian, black/African American non-Hispanic, Hispanic any race, and other. Individuals were asked if they were male or female. Income was assessed by household yearly income before taxes with the following response categories: None, under US $9999 per year, US $10,000 to US $29,999 per year, US $30,000 to US $49,999 per year, US $50,000 to US $69,999 per year, US $70,000 to US $89,999 per year, and US $90,000 or more per year. Education level was asked using the question *How many years of school have you completed*? *(check all that apply)* with associated response categories: Some high school, but did not graduate, high school graduate or General Educational Development (GED), some college, 2-year college graduate (eg, community college), 4-year college graduate, some graduate school or postgraduate degree, and trade school. Number in household was determined using the question *How many people (including yourself) currently live in the home where you live?* with the following response categories: 1, 2-4, and 5 or more. Employment status was assessed using the question *Which of the following best describes your current status? (check all that apply)* with the following response categories: employed full-time, employed part-time, in the military, unemployed and looking for work, unemployed and not looking for work, retired, student, homemaker, disabled or too ill to work, living off the land/a subsistence lifestyle (hunting, fishing, and berry gathering), and other.

#### Use, Satisfaction, and Perceived Effectiveness Questionnaire

At 6 weeks and 12 weeks, participants completed 12 multiple choice and 2 open-ended questions with no time frame specified. Use was assessed by asking a frequency question *About how often did you access the Health is Our Tradition: Balance and Harmony After Trauma website?* with response categories: more than once daily, once daily, 2-3 times a week, once a week, less than once a week, and never. Website use duration was assessed by the survey question *On average, how much time did you spend using the website once you accessed it?* with response categories: never went on the website, 5 min or less, at least 10 min, at least 30 min, at least 1 hour, more than 1 hour, and can’t recall. Barriers to use were assessed by asking *Did you experience any of the following difficulties using the website?* with response categories: I did not have access to a computer, my computer was not working, I forgot my password, I could not play the videos, I could not play the audio, I could not print, and other problems. Satisfaction was assessed by asking, *Overall, how satisfied are you with your experiences with Health is Our Tradition: Balance and Harmony after Trauma?* Responses to this item were: never went on the website, quite dissatisfied, indifferent or mildly satisfied, mostly satisfied, or very satisfied. Participants were asked if they would recommend the website to a friend with response categories: yes, definitely; yes, I think so; no, I don’t think so; and no, definitely not. Participants were asked if they would use the website in the future in the same manner. The first perceived effectiveness item was *If you completed at least one website Guide, did it help you deal more effectively with your traumatic experience and its consequences?* Response categories to this item were: yes, it helped a great deal; yes, it helped; no, it really didn’t help; and no, it seemed to make things worse. Perceived helpfulness of the text messages and testimonial videos were assessed in the same manner. Whether the testimonial videos were upsetting was also rated as: no, not upsetting; yes, slightly upsetting; yes, moderately upsetting; and yes, very upsetting.

#### Website Metrics

Employing industry-standard website analytic tools [46], we tracked visit patterns, frequency of use, and session duration. Types of interaction with each section (guide) in the website were measured (eg, advanced through an entire guide, played a video, entered personal data into an activity, completed a drag and drop activity, and completed a relaxation meditation) per individual.

#### Experience With Technology Questionnaire

This 16-item questionnaire, developed by the research team, assesses perceived skill and confidence using computers and peripherals as well as aspects of using the internet such as navigating between pages, installing software such as a flash player, managing popups, and downloading, saving, or printing files from the internet. Items assessing skills used the following stems *Can you…* or *Do you…* with binary (no/yes) response categories. Items assessing confidence of technology use had the following response categories: not confident at all; I usually need help; it takes me a while, but I can manage; and confident.

#### Posttraumatic Stress Disorder Screening

The PC-PTSD scale consists of 4 binary (no/yes) items assessing PTSD symptoms and included an introductory sentence to cue respondents to lifetime traumatic events. A positive screen is indicated and has been validated in primary care settings, when the sum of positive responses is 3 or higher [39].

#### Posttraumatic Stress Disorder Checklist—Civilian Version

The PCL-C assesses key symptoms of PTSD via 17 items on a 5-point rating scale: (1) not at all; (2) a little bit; (3) moderately; (4) quite a bit; or (5) extremely. The PCL-C is applied generally to any traumatic event with respondents considering how much they have been bothered by the PTSD symptom in the last month. Higher scores indicated more severe symptomatology.

#### Alcohol Use Disorders Identification Test

The AUDIT is a 10-item screener for alcohol use in the past year. Higher scores indicated higher levels of alcohol use and problems related to alcohol abuse. Possible responses included never (0), monthly (1), 2-4 times per month (2), 2-3 times a week (3), or 4 or more times a week (4).

#### Patient Health Questionnaire

The PHQ measures the severity of somatic, depression, and anxiety symptoms in the past 2 weeks. All items used rating scales. Somatic symptom severity was derived from the first 15 items (PHQ-15). Possible responses to these were not bothered (0), bothered a little (1), or bothered a lot (2). PHQ-15 scores of 5, 10, and 15 represented cut-points for low, medium, and high somatic symptom severity, respectively. The next 9 items (PHQ-9) measured depression symptoms with a 4-point rating scale: not at all (0), several days (1), more than half the days (2), nearly every day (3). These were followed by binary (no/yes) questions and 3-point ratings of frequency of anxiety symptoms: not at all (0), several days (1), and more than half the days (2). A final item assessed the severity of somatic, anxiety, and depression symptoms with possible responses of not difficult at all (0), somewhat difficult (1), very difficult (2), or extremely difficult (3). Higher scores indicated more severe anxiety/depression symptomatology.

#### Drug Abuse Screening Tool

The DAST is a 10-item screener for drug use in the past 12 months. Items were binary (no/yes); higher scores indicated higher levels of problems related to drug abuse.

#### American Indian Symptom Inventory

The American Indian Symptom Inventory was developed for this study [5], specifically for use with AI/AN people. Drawing on items from such common measures as the Symptom Checklist-90 [47], as well as items suggested by focus groups as highly relevant to psychological distress among AI/ANs, this 50-item measure assesses a wide range of symptomatology prefaced by the statement, “Here is a list of problems people may have. How much have any of these problems bothered or upset you during the past month, including today...Not at all (0), Some (1), or A lot (2).”

#### Electronic Health Records

Electronic health record data collected included date of birth to calculate age; any depression and substance use screening scores; diagnoses of behavioral and trauma-related physical health disorders (eg, International Classification of Diseases–10 codes); prescribed behavioral health medications; and number of visits to primary care, emergency room/urgent care, inpatient, other clinical/ambulatory, and behavioral health clinics.

### Data Analysis

Using SAS version 9.4 (SAS Institute, Inc), analysts created dummy variables for *check all that apply* questions (race, education, and employment) and examined frequencies, means, and standard deviations for these demographic responses as well as sex, number in household, and income. Chi-square, Fisher exact, or *t* tests were calculated to compare participant demographic characteristics by site. Changes in use, feasibility, and perceived effectiveness between the 6-week and 12-week follow-ups were examined with paired *t* tests. To examine the potential relationship of the intervention with service utilization, analysts compared clinic visit frequency from the health record data in the 12 months before the intervention (divided by 4 for comparison) and the 3 months after it with paired *t* tests. Patterns of symptomatology over the 12-week intervention was explored through one-way analysis of variances for repeated measures or repeated measures logistic regression tests. The Wilcoxon signed rank test for nonparametric data was used to test significance for nonnormally distributed data.

## Results

### Participant Demographics

A total of 24 participants were enrolled in the feasibility pilot at baseline (Table 3). Their mean age was 49 (SD 14) years, and 71% (17/24) were women. Participant data from SCF (n=12) and CNHS (n=12) were compared. The 2 samples were similar in most regards, except CNHS participants were more likely to have attained a 4-year college degree or greater (*P*=.04; Fisher exact Test), and be employed full time or part time (Χ^2^_1_=4.2; *P*=.04). One individual at each site was lost to follow-up before the 6-week measures resulting in a retention rate of 92% (22/24).

**Table 3:** Pilot participant demographics (N=24).

Characteristics		Value
Women, n (%)		17 (71)
Age (years), mean (SD)		49 (14)
**Education, n (%)**		
	Less than high school/completed high school and/or GED^a^	8 (33)
	Trade school/some college/2-year college graduate	8 (33)
	4-year college graduate/some graduate school/postgraduate degree	8 (33)
**Employment, n (%)**		
	Employed (full time, part time)	11(46)
	Military	0 (0)
	Unemployed	6 (25)
	Retired/student/homemaker/subsistence/disabled/too ill	7 (29)
**Pretax income, n (%)**		
	<US $10,000	8 (33)
	US $10,000-US $29,999	6 (25)
	US $30,000- US $49,999	5 (21)
	>US $50,000	4 (17)
**Number of household members, n (%)**		
	1	7 (29)
	2-4	12 (50)
	>5	5 (21)

^a^GED: General Educational Development.

### Intervention Use, Satisfaction, and Perceived Effectiveness

The majority of participants 86% (19/24) reported any use of the website at 6 weeks, and nearly all 91% (20/22) used it at 12 weeks. However, participants did not use the website with the intensity requested by the researchers. Twelve of 22 participants reported that they used the website at the recommended intensity (a minimum of 3 times per week) at 6 weeks. At 12 weeks, use at recommended intensity dropped to 36% (8/22) (Table 4). Twenty of 22 participants reported reading the weekly tip text messages at 6 weeks. Of these 20 participants, 75% (15/20) considered them as at least moderately helpful. Similarly, 96% (21/22) reported reading the messages at 12 weeks, and 91% (19/22) considered them at least moderately helpful. At both 6 and 12 weeks, the majority of participants were mostly or very satisfied with the website, would recommend it to a friend in need, and reported that the tools in the website helped them cope with their trauma and its consequences.

Testimonial videos were viewed by 77% (17/22) at 6 weeks and 82% (18/22) reported viewing at 12 weeks. In all, 77% (13/17) and 83% (15/18) reported feeling that the testimonials were at least moderately helpful at 6 and 12 weeks, respectively. Out of 17 participants, 4 (24%) noted that testimonial videos were moderately to very upsetting at 6 weeks, a rate that dropped to 0% at 12 weeks (*t*_14_=2.36; *P*=.03). Out of 22 participants, 20 (91%) reported at each time point that they would use the website again in the future. Difficulties in using the website included loss of the website address and/or password and slow internet connection speeds. One participant reported computer access/performance issues. A research team member spoke with the participant in the first week of the intervention and was able to address these issues, allowing this participant to use the website.

**Table 4:** Feasibility at 6 weeks and 12 weeks following enrollment.

Feasibility question			6 weeks (N=22)^a^, n (%)	12 weeks (N=22)^a^, n (%)
**Use**				
	**How often did you access the website?**			
		Never	3 (14)	2 (9)
		Less than once a week	3 (14)	4 (18)
		Once a week	4 (18)	8 (36)
		2-3 times weekly	9 (41)	6 (27)
		Once daily	2 (9)	1 (5)
		More than once daily	1 (5)	1 (5)
	**How much time was spent on the website each session?**			
		At least 10 min	9 (41)	8 (36)
		At least 30 min	3 (14)	7 (32)
		At least 1 hour	6 (27)	1 (5)
		More than 1 hour	1 (5)	4 (18)
		Can’t recall	0 (0)	1 (5)
	**Did you read the weekly tips sent to your email address or by text to your phone?**			
		Yes, read weekly tip texts/emails	20 (91)	21(96)
	**Did you use the audio option on any of the information screens or activities?**			
		Yes, used the audio option for any guide	13 (59)	13 (59)
	**Did you view any of the testimonial (real life story) videos?**			
		Yes, viewed 1+ testimonial videos	17 (77)	18 (82)
	**What difficulties did you experience using the website?**			
		No computer access	0 (0)	1 (5)
		Computer not working	1 (5)	1 (5)
		Forgot password	2 (9)	0 (0)
		Could not play the videos	0 (0)	1 (5)
		Could not play the audio	0 (0)	1 (5)
		Could not print	2 (9)	1 (5)
		Lost or confused about website address	3 (14)	1 (5)
		Wanted to use it on my smartphone but it didn’t work	2 (9)	2 (9)
**Satisfaction**				
	**How satisfied are you with your experiences with website?**			
		Never went on site	3 (14)	1 (5)
		Quite dissatisfied	1 (5)	1 (5)
		Indifferent or mildly satisfied	3 (14)	0 (0)
		Mostly satisfied	6 (27)	8(36)
		Very satisfied	9 (41)	12 (55)
	**Would you use the website again in the future?**			
		Yes, definitely	12 (55)	10 (46)
		Yes, I think so	8 (36)	10 (46)
		No, I don’t think so	1 (5)	2 (9)
		No, definitely not	0 (0)	0 (0)

	**Would you recommend the website to a friend in need?**			
		Yes, definitely	13 (59)	13 (59)
		Yes, I think so	7 (32)	9 (41)
		No, I don’t think so	1 (5)	0 (0)
		No, definitely not	0 (0)	0 (0)
**Perceived effectiveness**				
	**Did the website help you deal more effectively with your traumatic experience and its consequences?**			
		Yes, a great deal	7 (32)	6 (27)
		Yes, it helped	11 (50)	16 (73)
		No, it really didn’t help	1 (5)	0 (0)
		No, it seemed to make things worse	0 (0)	0 (0)
	**How helpful did you find the testimonial videos?**			
		Did not watch	5 (24)	4 (18)
		Not at all helpful	0 (0)	0 (0)
		Slightly helpful	3 (14)	3 (14)
		Moderately helpful	4 (18)	3 (14)
		Very helpful	9 (41)	12 (55)
	**Did you find any of the testimonial videos upsetting?^b^**			
		Did not watch	5 (24)	4 (18)
		No, not upsetting	9 (41)	13 (59)
		Yes, slightly upsetting	3 (14)	5 (23)
		Yes, moderately upsetting	2 (9)	0 (0)
		Yes, very upsetting	2 (9)	0 (0)
	**Helpfulness of weekly tip texts/emails**			
		Not at all helpful	2 (9)	0 (0)
		Slightly helpful	3 (14)	3 (14)
		Moderately helpful	11 (50)	10 (46)
		Very helpful	4 (18)	9 (41)

^a^A total of 24 participants were consented and enrolled, but 2 were lost to follow-up before the 6-week measures.

^b^Participants reported significantly lower levels of finding testimonial videos upsetting at 12 weeks compared with 6 weeks (*t*_14_=2.36; *P*=.03).

### Website Usage

A total of 158 unique website sessions were recorded in the 12-week study period. The average duration of a session per participant was 16 min. Although all guides in the website were completed at least once, some were completed by more participants (Table 5). The 4 guides labeled for *first time visitors* were completed at rates ranging from 42% (10/24 participants; What can you do for yourself while healing?) to 100% (24/24 participants; What is trauma?). All 4 *first time visitors* guides were only psychoeducational in nature and did not include interactive activities.

Among those for *returning visitors*, guides that did not include written exercises (*Be in the moment, Discover your story, Notice your thoughts and feelings, Relax through breathing, Relax through visualization*, and *Relax your body*) were completed by a minimum of 3 out of 24 participants (13%) and a maximum of 18 out of 24 participants (75%; Be in the moment). Guides that included written exercises (*Change feelings by changing thoughts, Change negative thinking patterns, Cope with trauma reminders, Develop healthy relationships, Express your feelings and wants*, and *Identify your values and goals*) were completed less frequently. The least frequently completed guide was Develop healthy relationships. Out of 24, 7 participants (29%) completed it. The most frequently completed guide was *Discover your story*, which was completed by 11 out of 24 participants (49%). Overall, completion rates among guides that were only psychoeducational in nature were greater (*t*_18_=−2.90; *P*<.01) than those that were interactive (guided relaxation, checklists/click or drag activities, and written exercises). There were no significant differences in completion rates relative to the content length of the guide.

**Table 5:** Health is Our Tradition guides (N=24).

Guide name^a^	Guide completion, n (%)
Trauma and your health: what is trauma?	21 (88)
Trauma and your health: how to get back in harmony?	11 (46)
Be in the moment	11 (46)
Trauma and your health: how trauma affects health?	10 (42)
Trauma and your health: common reactions to trauma	9 (38)
Notice your thoughts and feelings	9 (38)
Trauma and your health: what to do to feel better	8 (33)
Change how you think about sleep	8 (33)
Change negative thinking patterns	7 (29)
Identify your values and goals	7 (29)
Cope with trauma reminders	6 (25)
Plan something fun	6 (25)
Change feelings by changing thoughts	5 (21)
Relax through visualization	5 (21)
Discover your story	5 (21)
Form good sleep habits	5 (21)
Express your feelings and wants	4 (17)
Relax through breathing	4 (17)
Relax your body	3 (13)
Develop healthy relationships	2 (8)

^a^Listed by greatest number of guide completions.

### Feasibility of Measures and Indicators of Effectiveness

Active participants at both sites were able to complete all self-report measures at baseline, 6 weeks and 12 weeks. Analyses of patterns of symptoms found reductions in self-reported PTSD, depression/anxiety/panic, physical symptoms related to PTSD, and problematic alcohol use between baseline and the 12-week follow-up. Depression symptom severity decreased between baseline and the 12-week follow-up visit.

Both sites were also able to query all data elements from electronic health records. There were no significant changes in patterns of diagnoses, screening, medications, or service utilization during exposure to the website.

## Discussion

### Principal Findings

A culturally adapted website for AI/AN people reporting symptoms of PTSD was developed and assessed for feasibility in 2 large AI/AN primary care settings. The website was well received with no difference in use, satisfaction, perceived effectiveness, or technical skills needed for use by age or gender. However, only half of the participants reported using the website at the recommended intensity at the 6-week follow-up and just over one-third did so at the 12-week follow-up (Table 4). Although website usage was only moderate and decreased across the study period, participants reported that the website was helpful in coping with their PTSD symptoms and related problems, and some self-reported symptoms decreased between enrollment and 12 weeks. Leader and provider concerns voiced during Phase 1 interviews and focus groups that participants may be unduly triggered by the website content, clinically decompensate, or have other negative impacts from using the website on their own were not supported by the data.

Collecting measures via self-report and electronic health record queries was successful, and a larger effectiveness trial is also warranted and appears feasible. Our participant attrition of 8% (2/24) is notably lower than the sizable attrition rates reported in self-administered internet interventions for depression, anxiety, and PTSD [48,49]. The weekly text-messaged *tips* may have contributed to retention and intensity of use by reminding participants about the study, their follow-up appointments, and highlighting content that may have sparked their interest. Although internet connectivity and speed were concerns for this population, very few participants reported these items as barriers to website access and usage. Participant website usage occurred throughout the 12-week intervention period with psychoeducational and shorter interactive activities being completed by more participants compared with worksheet-based activities requiring data input by participants. These findings align with preliminary evaluation of the *PTSD Coach Online* app, where participants indicated psychoeducation and self-management components as moderately helpful with symptom self-management as the most useful functions of the intervention [37].

### Limitations

As a small pilot study with a convenience sample intended to assess feasibility, we cannot determine whether the culturally adapted intervention is effective in reducing posttraumatic and related symptoms. Key limitations of this study include its small sample size, lack of a control group, and lack of clinician-administered outcome measures. Calculations of website use via Google Analytics was limited as we could not link individuals to specific usage data owing to confidentiality and feasibility constraints. Text message reminder delivery, reading, and potential impacts on website usage were not electronically tracked. Finally, in the review of the AUDIT and DAST measures, we note that these measures assessed past year use rather than current use. Thus, we recommend that these measures be edited or reconsidered in a future effectiveness study. Although not many participants found the video testimonials upsetting, additional exploration of the degree of upset and content provoking upset is indicated. Intensity of use required for maximum therapeutic benefit should be assessed as well as potential order effects. The last guide was specifically developed, given feedback from Phase 1 participants about how trauma impacts important relationships, yet viewed by only 2 participants. Future research may be enhanced by adding clinician support to compare the effectiveness of the self-management WBTI alone compared with clinician-supported use. Additional usage log data collection and analysis methods may also be employed to assess more detailed activity of participant website usage behavior. Assessment of the effectiveness of text message reminders to reinforce website usage and effectiveness is warranted.

### Conclusions

Despite these challenges and limitations, the results indicate Web-based therapeutic support of AI/AN adults with posttraumatic symptoms is feasible and warrant a large-scale randomized control trial to assess its potential effectiveness. The website appears to be a promising intervention given feasibility testing in 2 large, real-world AI/AN health systems. The intervention could be easily implemented by behavioral health staff integrated into primary care settings and was satisfactory to and viewed as helpful by AI/AN adults in this study.

## Abbreviations

AI/AN: American Indian and Alaska Native
AUDIT: Alcohol Use Disorders Identification Test
CNHS: Cherokee Nation Health Services
DAST: Drug Abuse Screening Tool
FWWI: Family Wellness Warriors Initiative
GED: General Educational Development
HIT: health information technologies
PCL-C: PTSD Checklist—Civilian Version
PC-PTSD: Primary Care—PTSD Screen
PHQ-9: Patient Health Questionnaire depression scale
PTSD: Posttraumatic stress disorder
SCF: Southcentral Foundation
UCD: University of Colorado Denver
WBTI: Web-based therapeutic intervention
